# Breakdown of coevolution between symbiotic bacteria *Wolbachia* and their filarial hosts

**DOI:** 10.7717/peerj.1840

**Published:** 2016-03-28

**Authors:** Emilie Lefoulon, Odile Bain, Benjamin L. Makepeace, Cyrille d’Haese, Shigehiko Uni, Coralie Martin, Laurent Gavotte

**Affiliations:** 1 UMR7245, MCAM, Museum national d’Histoire naturelle, Paris, France; 2 Institute of Infection and Global Health, University of Liverpool, Liverpool, United Kingdom; 3 UMR7179 MECADEV, Museum national d’Histoire naturelle, Paris, France; 4 Institute of Biological Sciences, University of Malaya, Kuala Lumpur, Malaysia; 5 UMR5554 ISEM, Université de Montpellier II, Montpellier, France

**Keywords:** Symbiosis, Horizontal transmission, Cophylogenetic analysis, *Wolbachia*, Coevolution, Filarial nematodes

## Abstract

*Wolbachia* is an alpha-proteobacterial symbiont widely distributed in arthropods. Since the identification of *Wolbachia* in certain animal-parasitic nematodes (the Onchocercidae or filariae), the relationship between arthropod and nematode *Wolbachia* has attracted great interest. The obligate symbiosis in filariae, which renders infected species susceptible to antibiotic chemotherapy, was held to be distinct from the *Wolbachia*-arthropod relationship, typified by reproductive parasitism. While co-evolutionary signatures in *Wolbachia*-arthropod symbioses are generally weak, reflecting horizontal transmission events, strict co-evolution between filariae and *Wolbachia* has been reported previously. However, the absence of close outgroups for phylogenetic studies prevented the determination of which host group originally acquired *Wolbachia*. Here, we present the largest co-phylogenetic analysis of *Wolbachia* in filariae performed to date including: (i) a screening and an updated phylogeny of *Wolbachia*; (ii) a co-phylogenetic analysis; and (iii) a hypothesis on the acquisition of *Wolbachia* infection. First, our results show a general overestimation of *Wolbachia* occurrence and support the hypothesis of an ancestral absence of infection in the nematode phylum. The accuracy of supergroup J is also underlined. Second, although a global pattern of coevolution remains, the signal is derived predominantly from filarial clades associated with *Wolbachia* in supergroups C and J. In other filarial clades, harbouring *Wolbachia* supergroups D and F, horizontal acquisitions and secondary losses are common. Finally, our results suggest that supergroup C is the basal *Wolbachia* clade within the Ecdysozoa. This hypothesis on the origin of *Wolbachia* would change drastically our understanding of *Wolbachia* evolution.

## Introduction

*Wolbachia* (Rickettsiales, *Anaplasmataceae*) are *α*-proteobacteria closely related to *Ehrlichia* and *Anaplasma* species. They are widespread symbionts detected in all arthropod classes (infecting between 20 and 70% of insect species) (Bourtzis & Miller, 2003; Zug & Hammerstein, 2012) as well as in a nematode family, the Onchocercidae, commonly known as filariae (Bandi et al., 1998; Sironi et al., 1995). Thus, *Wolbachia* are present in millions of species. They are vertically transmitted from females to offspring. In addition, this bacterium induces a large range of phenotypes in its hosts, varying from mutualism, including obligate dependencies for reproduction and long-term survival (mostly described in filariae and some arthropods) (Hosokawa et al., 2010; Zug & Hammerstein, 2015; Hoerauf et al., 2003); to parasitism by interfering with host reproduction (in arthropods only) (Werren, Baldo & Clark, 2008). The spread of *Wolbachia* via cytoplasmic incompatibility, male killing, feminization and induction of parthenogenesis can have a major impact on the evolution of arthropod host populations, and in some cases, may drive speciation (Rokas, 2000; Telschow et al., 2007).

Studies on *Wolbachia* are more largely focused on arthropods mainly due to: the higher incidence of *Wolbachia* in arthropods (e.g., a greater number of infected species), differences of accessibility (rearing and collecting are easier for arthropods) and also the relatively late identification of *Wolbachia* in filariae. Indeed, although intracellular bacterial organisms had been observed in filariae tissues decades ago (Kozek, 1971; Kozek, 1977; McLaren et al., 1975), they were only molecularly identified as closely related to *Wolbachia pipientis* in the mid-1990s (Sironi et al., 1995). The obligate nature of the relationship between *Wolbachia* and filariae has led to clinical trials of antibiotics for the treatment of filarial diseases of humans, including lymphatic filariasis and onchocerciasis (Bandi et al., 1999; Taylor et al., 2000; Taylor et al., 2012).

The different *Wolbachia* strains have been classified in major phylogenetic lineages named supergroups. Initially, the phylogeny of *Wolbachia* described two clades (named supergroups A and B) infecting only arthropods. Two other clades were added after the investigation of bacteria in filariae (supergroups C and D) (Bandi et al., 1998). From 1999 to 2015, a multiplication of supergroup classifications occurred, with 10 clades identified that exclusively infect arthropods (supergroups E, G, H, I, K, M, N, O, P and Q) (Augustinos et al., 2011; Bing et al., 2014; Bordenstein & Rosengaus, 2005; Czarnetzki & Tebbe, 2004; Dittmar & Whiting, 2004; Glowska et al., 2015; Ros et al., 2009; Rowley, Raven & McGraw, 2004; Vandekerckhove et al., 1999; Wang et al., 2014); one infecting filariae (supergroup J) (Casiraghi et al., 2004); one identified in the plant parasite nematode *Radopholus similis* (Tylenchida) (supergroup L) (Haegeman et al., 2009); and finally supergroup F, which is apparently unique in infecting both arthropods and filariae (Casiraghi et al., 2001b; Lo et al., 2002).

These phylogenetic analyses led to *Wolbachia* classification gradually increasing in complexity from single gene analysis (Sironi et al., 1995; Werren, Zhang & Guo, 1995) to multi-locus methods (Baldo et al., 2006b; Bordenstein et al., 2009; Casiraghi et al., 2001a; Casiraghi et al., 2005; Lefoulon et al., 2012). More recently, the complete sequencing of various *Wolbachia* genomes has allowed the development of phylogenomic analyses (Comandatore et al., 2013; Fenn & Blaxter, 2006; Gerth et al., 2014; Nikoh et al., 2014; Ramirez-Puebla et al., 2015). The evolutionary history of *Wolbachia* has been investigated by using outgroups belonging to the *Anaplasma*, *Ehrlichia* or *Rickettsia* genera (O’Neill et al., 1992; Ramirez-Puebla et al., 2015) as an attempt to establish the origin of the bacteria. However, it has been suggested that these outgroups are too distant to the ingroups, and thus could introduce artefacts to the phylogenetic reconstruction (such as long-branch attraction, LBA), rendering it very challenging to determine the host group that initially acquired *Wolbachia* infection with confidence (Bordenstein et al., 2009).

The co-evolutionary pattern observed for *Wolbachia* varies between hosts (Bandi et al., 1998; Bourtzis & Miller, 2003). First, composite dynamics of acquisition and loss of bacteria strains in arthropods has made the picture very complex at a co-evolutionary scale (Baldo et al., 2008; Gerth, Rothe & Bleidorn, 2013; O’Neill et al., 1992; Vavre et al., 1999). Second, co-speciation of filariae and *Wolbachia* has been hypothesized (Bandi et al., 1998), even if several cases of secondary losses and two cases of horizontal transmission between species have been detected, suggesting this scenario is too simplistic (Ferri et al., 2011; Lefoulon et al., 2012; Martin & Gavotte, 2010). Moreover, to date, the notion of coevolution between *Wolbachia* and filariae was based on a poorly resolved evolutionary history of the Onchocercidae and limited sampling (just 10 examined species) (Bandi et al., 1998).

We have recently revised the evolutionary history of the Onchocercidae by completing the most comprehensive analysis to date, using a multi-gene phylogeny on 45 different species representing seven of the eight subfamilies (Lefoulon et al., 2015). Here, we propose a reappraisal of the coevolutionary pattern between *Wolbachia* and filariae using this significantly more intensive sampling. We identify new *Wolbachia* strains, critically examine the accuracy of the *Wolbachia* supergroups, reassess the prevalence of *Wolbachia* infection within the Onchocercidae and revisit the hypothesis of ancestral *Wolbachia* absence. Our co-phylogenetic analyses reveal multiple events of losses and acquisitions of the bacteria in the filariae, highlighting strong but localized patterns of co-evolution in some clades, in contrast with multiple horizontal transfers and a breakdown of co-evolution in others. Finally, we apply a new approach to the question of initial acquisition of *Wolbachia* within the Ecdysozoa.

## Materials and Methods

### Specimens

DNA from 45 filariae, including 2 outgroup species that fall outside the Onchocercidae (obtained from Lefoulon et al. (2015)), as well as 5 specimens of arthropods (Tables 1 and 2), were used for molecular analyses. In addition, some filarial specimens were used for histological analyses (Table 1). All procedures were conducted in compliance with the rules and regulations of the respective competent national ethical bodies (Table S1) (Lefoulon et al., 2015). Some non-human vertebrates were captured for experimental procedures, subject to the ethics approval of the relevant national bodies, while others were obtained at abattoirs or donated to the MNHN by hunters or veterinarians (Table S1). The MNHN does neither solicit nor compensate for these donations. Nematode accession numbers are reported in Table 1. For the author(s) and year of parasite and host species collection, the reader is referred to File S1. All the samples were fixed and kept in 70% ethanol. For nematodes, the median parts were used for molecular analyses; the anterior and posterior parts being retained for possible morphological analyses.

**Table 1:** Details on filariae and other nematodes for which new molecular and/or histological analyses were performed. The first two columns indicate the subfamilies and the names of the species. For the author(s) and year of collection of each parasite and host species, the reader is referred to File S1. The last three columns indicate respectively: host species; Muséum National d’Histoire Naturelle Paris registration number; and country of specimen collection.

Subfamilies	Species	Host	No.	Collection place
Oswaldofilariinae	*Oswaldofilaria chabaudi*	*Tropidurus torquatus*	191YU	Brasil
	*Oswaldofilaria petersi*	*Crocodilurus amazonicus*	34PF	Peru
Waltonellinae	*Ochoterenella* sp.1	*Rhinella granulosa*	200YU	Venezuela
	*Ochoterenella* sp.2	*Rhinella marina*	47YT	Venezuela
	*Ochoterenella* sp3	*Phyllomedusa bicolor*	194JW	French Guyana
Icosiellinae	*Icosiella neglecta*	*Rana ridibunda*	44YT	Ukraine
		*Rana esculeta*	45YT	France
Setariinae	*Setaria labiatopapillosa*	*Bos taurus*	413YU	Cameroon
	*Setaria tundra*	*Rangifer tarandus*	71YT	Finlande
Dirofilariinae	*Dirofilaria* (*Dirofilaria*) *immitis*	*Canis familiaris*	79YT	ES
	*Dirofilaria (Nochtiella) repens*	*Canis familiaris*	297YU	Italy
	*Foleyella candezei*	*Agama agama*	68CE	Togo
	*Loa loa*	*Homo sapiens*	80YT	France
	*Pelecitus fulicaeatrae*	*Podiceps nigricollis*	49YT	Spain
Splendidofilariinae	*Aproctella alessandroi*	*Saltator similis*	117YU	Brasil
	*Cardiofilaria pavlovskyi*	*Oriolus oriolus*	180YU	Bulgaria
	*Madathamugadia hiepei*	*Pachycactylus turneri*	81YU	South Africa
	*Rumenfilaria andersoni*	*Rangifer tarandus*	94YU	Finlande
Onchocercinae	*Acanthocheilonema odendhali*	*Callorhinus ursinus*	401YU	Alaska
	*Acanthocheilonema viteae*	*Meriones unguiculatus*	7YT	ES
	*Breinlia* (*Breinlia*) *jittapalapongi*	*Rattus tanezumi*	78YT	Laos
	*Brugia malayi*	*Meriones unguiculatus*	8YT	ES
	*Brugia pahangi*	*Meriones unguiculatus*	46YT	ES
	*Brugia timori*	*Homo sapiens*	6YT	Indonesia
	*Cercopithifilaria bainae*	*Canis familiaris*	9YT	ES
	*Cercopithifilaria rugosicauda*	*Capreolus capreolus*	350YU	France
	*Cruorifilaria tuberocauda*	*Hydrochoerus hydrochaeris*	55YT	Venezuela
	*Dipetalonema caudispina*	*Ateles paniscus*	362YU	Guyana
		*Ateles* sp.	64YT	Guyana
	*Dipetalonema gracile*	*Cebus olivaceus*	124CV	Venezuela
		*Cebus apella*	215YU	Peru
		*Ateles* sp.	63YT	Guyana
	*Dipetalonema graciliformis*	*Saimiri scuireus*	220YU	Peru
	*Dipetalonema robini*	*Lagothrix poeppigii*	216YU	Peru
	*Litomosoides brasiliensis*	*Carollia perspicillata*	35/37PF	Peru
	*Litomosoides hamletti*	*glossophaga soricina*	36PF1	Peru
	*Litomosoides sigmodontis*	*Meriones unguiculatus*	186MS	ES
	*Litomosoides solarii*	*Trachops cirrhosus*	213YU	Venezuela
	*Loxodontofilaria caprini*	*Capricornis crispus*	YG2-58	Japan
	*Mansonella* (*Cutifilaria*) *perforata*	*Cervus nippon*	216JW	Japan
	*Mansonella* (*Mansonella*) *ozzardi*	*Homo sapiens*	77YT	Haiti
	*Monanema martini*	*Arvicanthis niloticus*	31NC	Senegal
	*Onchocerca armillata*	*Bos taurus*	54FK	Cameroon
	*Onchocerca dewittei japonica*	*S. scrofa leucomystax*	OB9	Japan
	*Onchocerca eberhardi*	*Cervus nippon*	S63-5	Japan
	*Onchocerca gutturosa*	*Bos taurus*	54FK	Cameroon
	*Onchocerca ochengi*	*Bos taurus*	54FK	Cameroon
	*Onchocerca skrjabini*	*Cervus nippon*	S63-6	Japan
	*Yatesia hydrochoerus*	*Hydrochoerus hydrochaeris*	52YT	Venezuela
Outgroups	*Filaria latala*	*Panthera leo*	62YT	South Africa
	*Protospirura muricola*	*Gorilla* sp.	97YU	Central African Republic

**Notes.**

Abbreviation ES Experimental strains

**Table 2:** Details of arthropods specimens for which new molecular analyses were performed. The first columns indicate the families and the name of the species. For the author(s) and collection year of specimens, the reader is referred to File S1. The two last columns indicate Muséum National d’Histoire Naturelle Paris registration number and country of specimen collection, respectively.

Families	Species	No. MNHN	Collection place
Cimicidae	*Cimex lectularius*	11YT	France
Drosophilidae	*Drosophila simulans*	394YU	France (experimental)
Encyrtidae	*Ixodiphagus hookeri*	383YU	France
Isotomidae	*Folsomia candida*	EA010816	France (experimental)
Termitidae	*Nasutitermes* sp.	86YT	Venezuela

### Molecular screening

DNA was extracted from nematodes or arthropods using the QIAamp kit following the recommended procedures of the manufacturer (Qiagen, France), with a preliminary step of disruption for 2 cycles of 30 s at 30 Hz using a TissueLyser II (Qiagen, Germany) and incubation at 56 °C with proteinase K overnight. The presence of *Wolbachia* was determined by nested PCR screening of the seven genes (16S rDNA gene, *dnaA*, *coxA*, *fbpA*, *gatB*, *ftsZ* and *groEL*) as described in Table S2. PCR products were purified using the SV Wizard PCR Purification Kit (Promega, USA) and directly sequenced. A total of 198 sequences were deposited in the GenBank Data Library: KU255197 to KU255395 (Table S3).

### Immunohistochemical staining of nematode sections

The presence or absence of *Wolbachia* was determined by immunohistochemical staining according to Kramer et al. (2003). A rabbit polyclonal antiserum raised against the *Wolbachia* surface protein (WSP) of *Wolbachia* from *Brugia pahangi* (Wol-Bp-WSP, dilution 1:2000 (designed by Bazzocchi et al. (2000) and provided by Dr. Maurizio Casiraghi) was used to stain 5 μm paraffin sections of filarial species placed on Superfrost Plus slides (Thermo Scientific) as described by Ferri et al. (2011). Sections of the laboratory strain *Litomosoides sigmodontis* were used as a positive control. Negative controls were carried out by omitting the primary antibody. In addition, transverse sections were stained with haematoxylin and eosin for the identification of anatomical structures.

### Phylogenetic reconstruction

The *Wolbachia* 16S rDNA, *dnaA*, *groEL*, *ftsZ*, *coxA, fbpA* and *gatB* sequences were aligned with sequences available in Genbank (Table S3) using Muscle (Edgar, 2004). To check for the absence of stop codons, the alignment of coding genes was translated using EMBOSS Transeq (Li et al., 2015), and a comparison with available transcript sequences was performed. 12 complete genomes or draft genomes of *Wolbachia* from arthropods and 5 complete or draft genomes of *Wolbachia* from filariae were used in the analyses (Table S3). *Wolbachia* from arthropods included in the analyses were selected based on the availability of the 7 genetic markers we used (with exception of *Wolbachia* from *O. horni*, *T. deion*, *Z. angusticollis* and *Z. nevadensis*). Presence of recombination events were evaluated by RDP (Martin & Rybicki, 2000), GENECONV (Padidam, Sawyer & Fauquet, 1999), BootScan (Salminen et al., 1995), MAXCHI (Smith, 1992), Chimaera (Posada & Crandall, 2001), SISCAN (Gibbs, Armstrong & Gibbs, 2000) and 3Sequ (Boni, Posada & Feldman, 2007) methods using RDP v4.38 (Martin et al., 2015). Tests of nucleotide substitution saturation were performed on alignments with and without outgroups by Xia’s method (Xia et al., 2003) using DAMBE version 5 (Xia & Xie, 2001). A supermatrix of these seven alignments was generated using Seaview (Gouy, Guindon & Gascuel, 2010). Two different nucleotide datasets were analysed for different purposes: one alignment of 64 *Wolbachia* without outgroups for the phylogenic analysis, and one alignment including only *Wolbachia* from filarial nematodes was studied for the cophylogenetic analysis. In addition, both a nucleotide and an amino-acid dataset of 54 *Wolbachia* strains, including five outgroups (*Anaplasma marginale* Theiler, 1910, *Anaplasma phagocytophilum* (Dumler et al., 2001), *Ehrlichia chaffeensis* Anderson et al., 1992, *Ehrlichia muris* Wen et al., 1995, *Ehrlichia ruminantium* (Dumler et al., 2001), were generated. Best-fitting evolutionary models according to a supermatrix were determined by JModelTest 2.1 (Darriba et al., 2012) or by ProtTest 2.4 (Abascal, Zardoya & Posada, 2005) using the corrected version of the Akaike Information Criterion (AICc). For the nucleotide supermatrix, the phylogenies of filariae and *Wolbachia* were performed by Maximum Likelihood (ML) inference using the model GTR+ I+Γ with RAxML version 8 (Stamatakis, 2014). For each gene, a partitioned model was implemented to estimate evolution parameters. The robustness of nodes was assessed with 1,000 bootstrap replicates. ML analysis of the amino acid supermatrix was performed using the model HIVb+ I+Γ + F with PhyML (Ronquist & Huelsenbeck, 2003). The robustness of nodes was assessed with 500 bootstrap replicates.

### Cophylogenetic analysis

Two methods were performed to study cophylogenetic patterns between filariae and their *Wolbachia* symbionts: a global-fit method and an event-based method.

The global-fit method estimates the congruence between two phylogenetic trees. The global fit of filarial phylogeny with their bacterial phylogeny was estimated using the PACo application (Balbuena, Miguez-Lozano & Blasco-Costa, 2013) in the R environment (R Core Team, 2013). This method is a distance-based test: briefly, the ML phylogenetic trees were transformed into matrices of pairwise patristic distance, then into matrices of principal coordinates (PCo). The PCo of *Wolbachia* were transformed by Procrustes analysis using least-squares superimposition to minimize the differences with filarial PCo. An ordination plot was produced, representing the Procrustean global fit. The global fit was evaluated by the residual sum of squares value (m${}_{XY}^{2}$) of the Procrustean fit calculation, which is inversely proportional to the topological congruence, and its significance was tested by random permutations (100,000,000 permutations). Each host-symbiont association was evaluated by a jackknife procedure (Sokal & Rohlf, 1981) to estimate the square residual of each single association and its 95% confidence interval. A bar chart plot of these jackknifed squared residual was produced. Low residuals are interpreted as a low contribution of m${}_{XY}^{2}$ and thus as a strong congruence between the filariae and the bacteria.

The event-based method explores co-evolutionary scenarios in order to find the best reconstructions by minimizing the overall cost, given a cost regime for evolutionary events (Charleston, 1998). Jane 4.0 (Conow et al., 2010) was used to associate overall costs of co-evolutionary scenarios between *Wolbachia* and their hosts. The default settings for cost regimes are: a “co-speciation” event (two partners speciate simultaneously) is associated with null cost; a “duplication” event (the symbionts speciate in the same host) and “loss” event (the symbiont does not speciate while the host does) are associated with a cost equal to one; and a “duplication then host switching” event (the symbiont speciates and one switches to another host) is associated with a cost equal to two (Charleston, 1998). All analyses were performed with a number of generation of 500 and a population of 30.

This method is only manageable on rooted phylogenies, yet rooting of *Wolbachia* phylogeny is contested due to the absence of appropriate outgroups (Bordenstein et al., 2009). In order to circumvent this problem, the estimation of the co-evolutionary scenario associated with the minimum overall cost was analysed using different rooting constraints on the *Wolbachia* phylogeny.

An initial analysis was performed including only filariae and their *Wolbachia* symbionts (62 hosts and 36 symbionts). This analysis was performed without constraint or with a time constraint. Although Jane’s program does not change topologies (i.e., the relationships between hosts) it can modify the distance branches (i.e., the nodes representing the events of speciation are not fixed by timescale) (Conow et al., 2010). Thus, without time constraint, “host switch” events are not limited by temporal scales and the algorithm can change branch length on the filarial tree to minimize scenario cost, at the risk that this is inconsistent with filarial evolution. Subsequently, two time zones were defined to provide constraints to indicate that the first speciation events leading to filariae belonging to ONC1, ONC2 and ONC3 (thus within time zone no. 1) occurred before the first speciation events leading to filariae belonging to ONC4 and ONC5 (thus within time zone no. 2) (Lefoulon et al., 2015). Thus, *Wolbachia* speciation events were constrained within the second time zone.

A second analysis was performed adding *Wolbachia* from arthropods (64 *Wolbachia* strains in total). This analysis was made possible because the Jane program manages topologies and not distance branches (Conow et al., 2010). A hypothetical arthropod evolutionary history based on previous studies (Legendre et al., 2015; Misof et al., 2014; Tong et al., 2015; Wiegmann et al., 2011) was added to the nematode topology in order to perform these analyses. This second analysis was conducted without time constraint because the timescale of evolution for all the hosts (arthropods and filariae) is poorly known. If time zones were defined, events of *Wolbachia* speciation should be constrained into these zones. The definition of these temporal constraints appears arbitrary so far as timescales are not determined.

## Results/Discussion

### Absence of *Wolbachia* in filariae: ancestral absence and secondary losses

**Figure 1:**
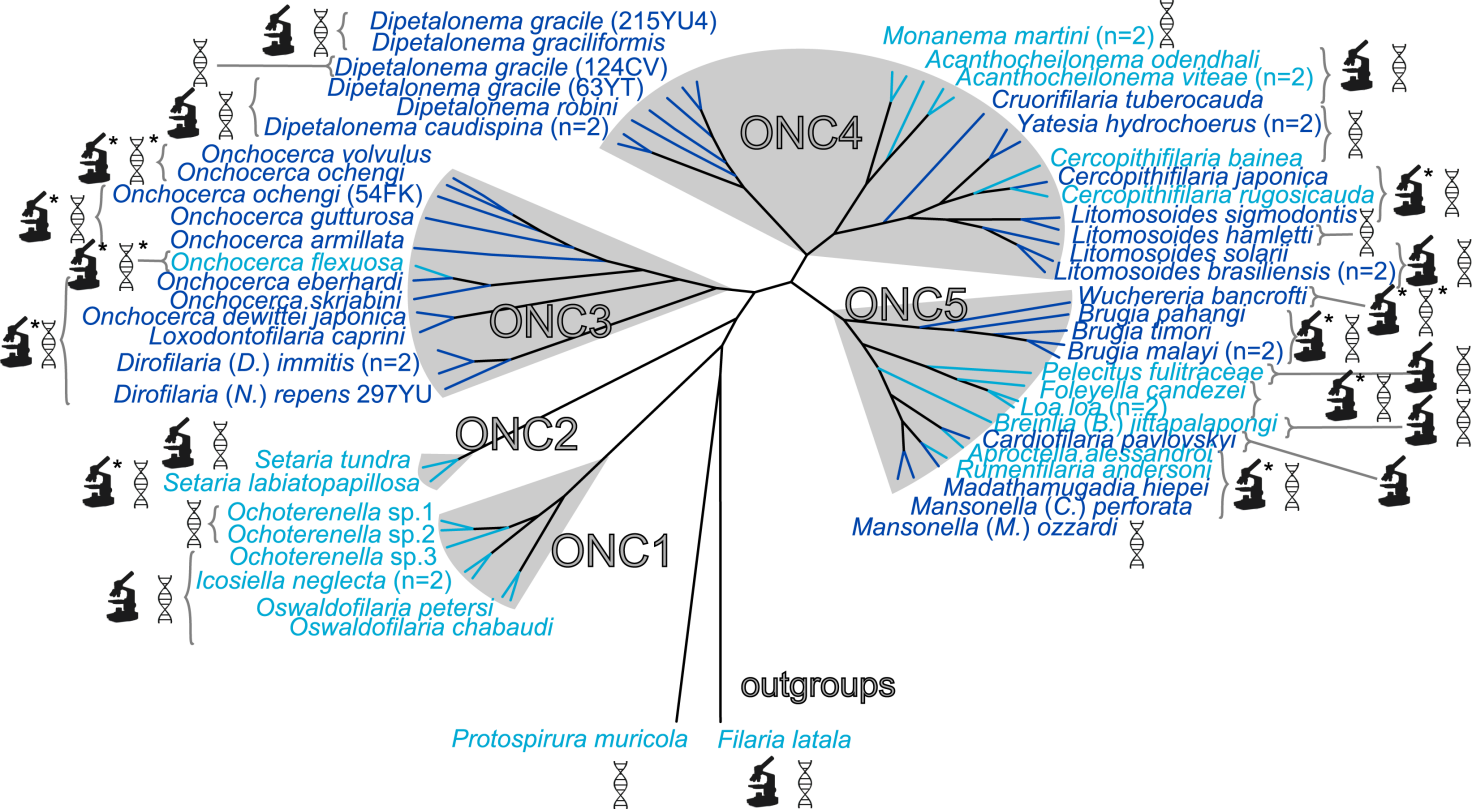
Presence/absence of *Wolbachia* in filariae (Onchocercidae). Sixty onchocercid specimens from 45 species were analysed. *Wolbachia* screening was performed by either PCR and/or immunohistostaining with a rabbit polyclonal antiserum against *Wolbachia* surface protein (WSP) of *Brugia pahangi Wolbachia* (Wol-Bp-WSP, dilution 1:2,000). Results of *Wolbachia* screening are indicated by colour: light blue for *Wolbachia*-free species and dark blue for *Wolbachia-*positive species. Detection methods for *Wolbachia* are illustrated next to the species name using specified symbols: DNA double helix symbol, DNA amplification by PCR; microscope symbol, immunohistostaining of WSP; ∗, determine by previous studies. The filarial clades (ONC1-ONC5) were defined according to Lefoulon et al. (2015). Phylogeny of Onchocercidae was based on partitioned concatenated datasets of 12S rDNA, *coxI*, *rbp1*, *hsp70*, *myoHC*, 18S rDNA, and 28S rDNA sequences using Maximum Likelihood Inference.

The detection of *Wolbachia* was performed on 16 new filarial species among a total of 45 examined species (Table 1). Nine of the 16 new species were *Wolbachia*-free (based on PCR screening and/or WSP antibody staining) (Fig. 1 and Table 3). The *Wolbachia* infection status (i.e., presence or absence) in the other species was in agreement with previous studies (Fig. 1). Absence of *Wolbachia* infection in filarial species could be explained by two hypotheses: either an ancestral absence or a secondary loss (Casiraghi et al., 2004).

**Table 3:** *Wolbachia* distribution in tissues of 20 filarial nematode specimens by immunostaining.

Species	MNHN no	Sex	LC	GL			Testes	SG	Intestine
				Oocyte	Egg	mf			
*Acanthocheilonema odendhali*	401YU	f	−	NA	NA	−	NA	−	−
*Breinlia* (*Breinlia*) *jittapalapongi*	78YT	m	−	NA	NA	NA	−	−	−
*Cardiofilaria pavlovskyi*	180YU	f	+	NA	+	NA	NA	−	+
*Cruorifilaria tuberocauda*	57YT	f	+	NA	NA	+	NA	−	−
*Cruorifilaria tuberocauda*	60YT	f	+	NA	NA	+	NA	−	−
*Dipetalonema caudispina*	64YT	f	+	NA	−	NA	NA	−	−
*Dipetalonema gracile*	215YU	f	+	NA	+	NA	NA	−	−
*Dipetalonema graciliformis*	220YU	f	+	NA	+	NA	NA	−	−
*Dipetalonema robini*	216YU	f	+	NA	+	NA	NA	−	−
*Icosiella neglecta*	122YU	f	−	−	NA	NA	NA	−	NA
*Litomosoides brasiliensis*	35PF	f	+	+	+	NA	NA	−	−
*Litomosoides solarii*	213YU	f	+	NA	+	NA	NA	−	−
*Ochoterenella phyllomedusa*	194JW	f	−	NA	−	−	NA	−	−
*Oswaldofilaria chabaudi*	102YU	f	NS	NA	−	NA	NA	−	NS
*Oswaldofilaria petersi*	34PF	f	−	−	NA	NA	NA	−	NA
*Pelecitus fulicaeatrae*	150YU	f	−	NA	−	−	NA	−	−
*Setaria tundra*	71YT	f	−	NA	−	NA	NA	−	NS

**Notes.**

Abbreviation LC lateral chords (hypodermis) GL germline mf microfilaria SG somatic germline + positive staining − no staining ns no specific staining NA not available due to position of the section

We have previously identified five strongly supported clades within the Onchocercidae that we have named ONC1 to ONC5 based on their monophyly (Fig. 1) (Lefoulon et al., 2015). The ancestrally derived species belonging to the genera *Oswaldofilaria*, *Icosiella* and *Ochoterenella* (clade ONC1) are not infected by *Wolbachia* (Fig. 1). This result supports, as previously suggested, the absence of *Wolbachia* infection as an ancestral trait (Bain et al., 2008; Ferri et al., 2011; Lefoulon et al., 2012). Absence of *Wolbachia* has also been noted in the other nematodes and nematomorph species examined so far (about 30 examined species) (Bordenstein, Fitch & Werren, 2003; Duron & Gavotte, 2007; Foster et al., 2014), with the exception of the plant parasitic nematode *Radopholus similis* (Haegeman et al., 2009), emphasizing the hypothesis of ancestral absence. Nevertheless, the hypothesis of secondary loss(es) cannot be ruled out, especially considering a recent discovery of *Wolbachia* genetic sequences within the host nuclear genome (“nuwts”, nuclear *Wolbachia* transfers) of the *Wolbachia*-free nematode *Dictyocaulus viviparus* (Rhabditina), which would imply secondary losses in at least some nematode clades outside the Onchocercidae (Koutsovoulos et al., 2014).

In more recently derived groups (clades ONC3, ONC4 and ONC5), infection was detected in a majority of species (71%) (Fig. 1). The more parsimonious hypothesis to explain the lack of infection in species belonging to these groups is a series of secondary losses, instead of a more costly scenario with multiple independent acquisitions. Thus, absence of infection in *Pelecitus fulicaeatrae*, *Acanthocheilonema odendhali*, *Breinlia* (*Breinlia*) *jittapalapongi* (ONC5) and *Cercopithifilaria bainae* (ONC4) are likely due to such losses. This hypothesis is strengthened by the identification of nuwts in *Wolbachia*-free species, such as *Onchocerca flexuosa* (ONC 3) and *Acanthocheilonema viteae* (ONC 4) (McNulty et al., 2010) and small *Wolbachia*-derived sequences (<500 bp) in *Loa loa* (ONC 5) (Desjardins et al., 2013).

### Occurrence of *Wolbachia* infection: where do we stand?

Among the 26 species harbouring a *Wolbachia* infection, seven new *Wolbachia* strains were identified (Fig. 1) in the following filarial species: *Cruorifilaria tuberocauda*, *Yatesia hydrochoerus*, *Dipetalonema caudispina*, *D. graciliformis*, *D. robini, Litomosoides solarii* (all in ONC4) and *Cardiofilaria pavlovskyi* (ONC5). Thus, *Wolbachia* infection occurs in 58% of our sample. Combining these data with those from former studies (Table S4), the occurrence of *Wolbachia* infection is 52.9% across 85 analysed filarial species. However, it is important to emphasise that most of the examined species belong to the more recently derived Onchocercinae subfamily, so the estimated occurrence of *Wolbachia* infection within the filariae still may be biased towards infected species (Fig. 1). For example, the filariae from birds, squamates and amphibians are largely understudied (Ferri et al., 2011). *Wolbachia* were typically present in the hypodermis of filariae and in the female germline (Table 3, Figs. S1 and S2), except for a specimen of *Cardiofilaria pavlovskyi*, in which an additional localisation in the intestinal wall cells was observed. This tissue distribution has also been reported for *Madathamugadia hiepei* (Lefoulon et al., 2012) and *Mansonella* (*Cutifilaria*) *perforata* (Ferri et al., 2011).

### Updated, unrooted *Wolbachia* phylogeny highlights accuracy of supergroup J

**Figure 2:**
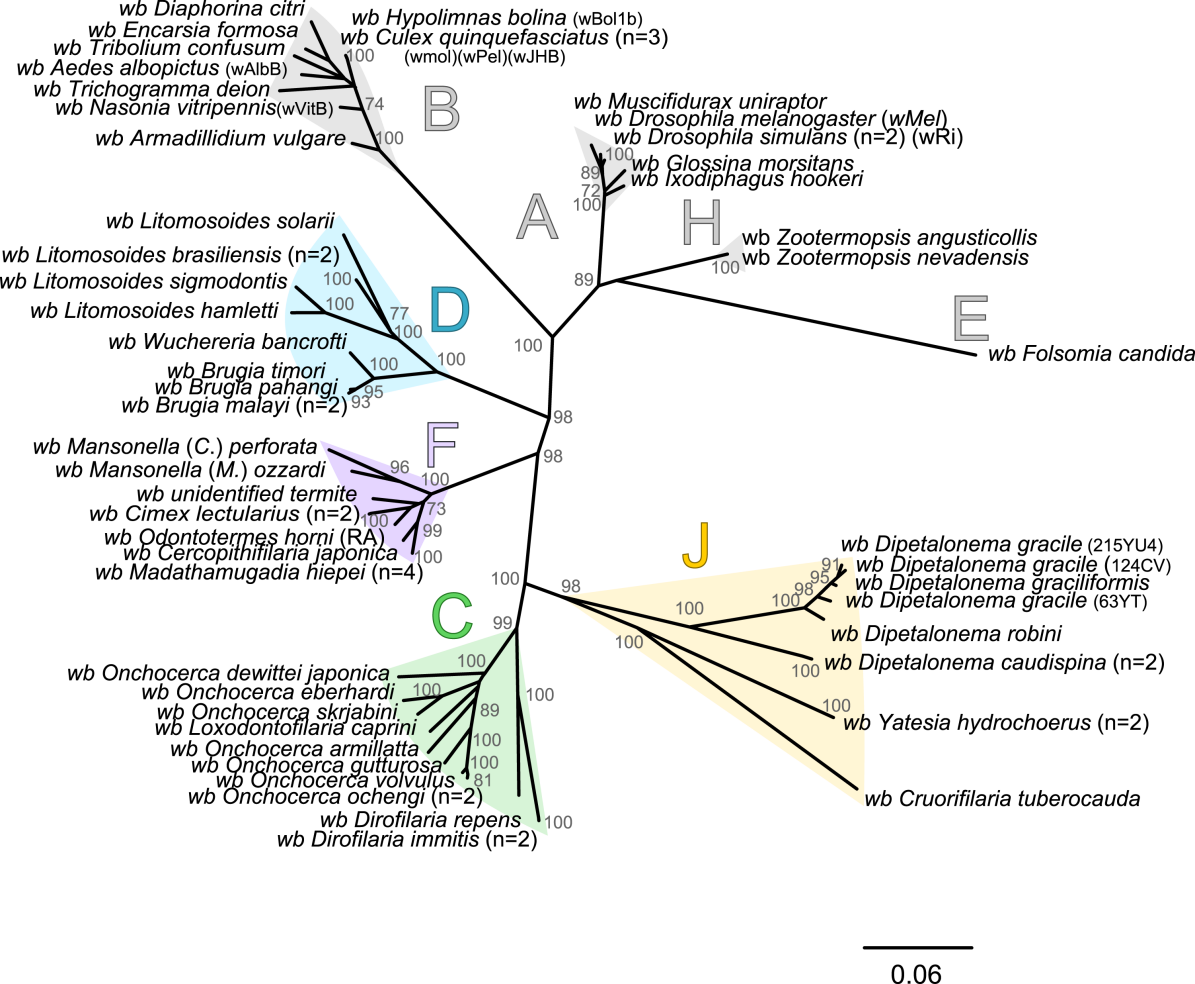
Phylogenetic tree of *Wolbachia* based on 7 markers by Maximum Likelihood. Analysis based on concatenation of 16S rDNA, *dnaA*, *groEL*, *ftsZ*, *coxA*, *fbpA* and *gatB*. The total length of datasets is approximately 4,600 bp. Sixty-four *Wolbachia* strains were analysed. The topology was inferred using Maximum Likelihood (ML) inference using RaxML. Nodes are associated with Bootstrap values based on 1,000 replicates. The *Wolbachia* supergroups (A–H) were identified according to Werren, Zhang & Guo (1995), Bandi et al. (1998), Lo et al. (2002) and Ros et al. (2009). The scale bar indicates the distance in substitutions per nucleotide. Abbreviation: *wb*, *Wolbachia*; n, number of studied specimens.

The present unrooted *Wolbachia* phylogeny includes 64 *Wolbachia* strains and is based on the concatenation of seven genes (16S rDNA, *groEL*, *ftsZ*, *dnaA*, *gatB*, *fbpA* and *coxA*) (Fig. 2). While no events of recombination were found within *coxA*, *gatB*, *ftsZ*, *dnaA* sequences, some were detected in 16S rDNA, *fbpA* and *groEL* sequences as presented in Table 4. These events occurred exclusively between supergroups A and B *Wolbachia*, as previously described (Baldo et al., 2006a). The resultant phylogeny reveals 7 strongly supported clades representing the seven previously defined supergroups A, B, C, D, F, H, and J (Bandi et al., 1998; Lo et al., 2002; Ros et al., 2009; Werren, Zhang & Guo, 1995).

**Table 4:** Detection of potential recombination events between *Wolbachia* sequences. Analyses of trace evidence of recombination events were performed using different methods as specified in the first column, and a *p*-value for their significance is indicated in parentheses. Regarding the trace evidence detected, no beginning breakpoint was identified.

Methods	16S rDNA	*coxA*	*gatB*	*ftsZ*	*dnaA*	*fbpA*	*groEL*
RDP	0	0	0	0	0	0	0
GENECONV	0	0	0	0	0	0	0
BootScan	0	0	0	0	0	0	1 (9, 12 × 10^−3^)
MaxChi	7 (1, 7161 × 10^−2^)	0	0	0	0	0	11(3, 35 × 10^−2^)
Chimaera	0	0	0	0	0	0	0
SiScan	3 (1, 909 × 10^−13^)	0	0	0	0	0	0
3Sequ	0	0	0	0	0	2 (8, 522 × 10^−3^)	0

*Wolbachia* from the *Dipetalonema* species (*D. gracile*, *D. caudispina*, *D. robini* and *D. graciliformis*) constitute a strongly supported group (Fig. 1). *Wolbachia* from *Yatesia hydrochoerus* and *Cruorifilaria tuberocauda* were closely related and are also linked to the *Wolbachia* from the *Dipetalonema* species. All together, they formed a strongly supported clade that confirm the validity of supergroup J (Ros et al., 2009), even if our results suggest two distinct J subgroups. However, we do not propose a new supergroup to prevent the multiplication of *Wolbachia* clades. We hold this position due to the following facts: the status of several supergroups is questionable because either they were described only on the basis of a few genetic markers (for example supergroups M and N infecting aphids (Augustinos et al., 2011; Bing et al., 2014)), or they contained only one or few host species such as supergroup J (Casiraghi et al., 2004; Ros et al., 2009). Up to now, *Wolbachia* in *Dipetalonema gracile* was the only representative of supergroup J. It was initially described as a deep branch within the supergroup C (Casiraghi et al., 2004), and then defined as a divergent lineage (Casiraghi et al., 2005; Ros et al., 2009). The accuracy of supergroup J was subsequently questioned, and its insertion into supergroup C has been proposed (Bordenstein et al., 2009; Glowska et al., 2015; Koutsovoulos et al., 2014). Our result clearly supports the accuracy of supergroup J and confirm its position as a sister group of supergroup C as previously suggested (Casiraghi et al., 2005).

### Coevolution of *Wolbachia* and Onchocercidae

**Figure 3:**
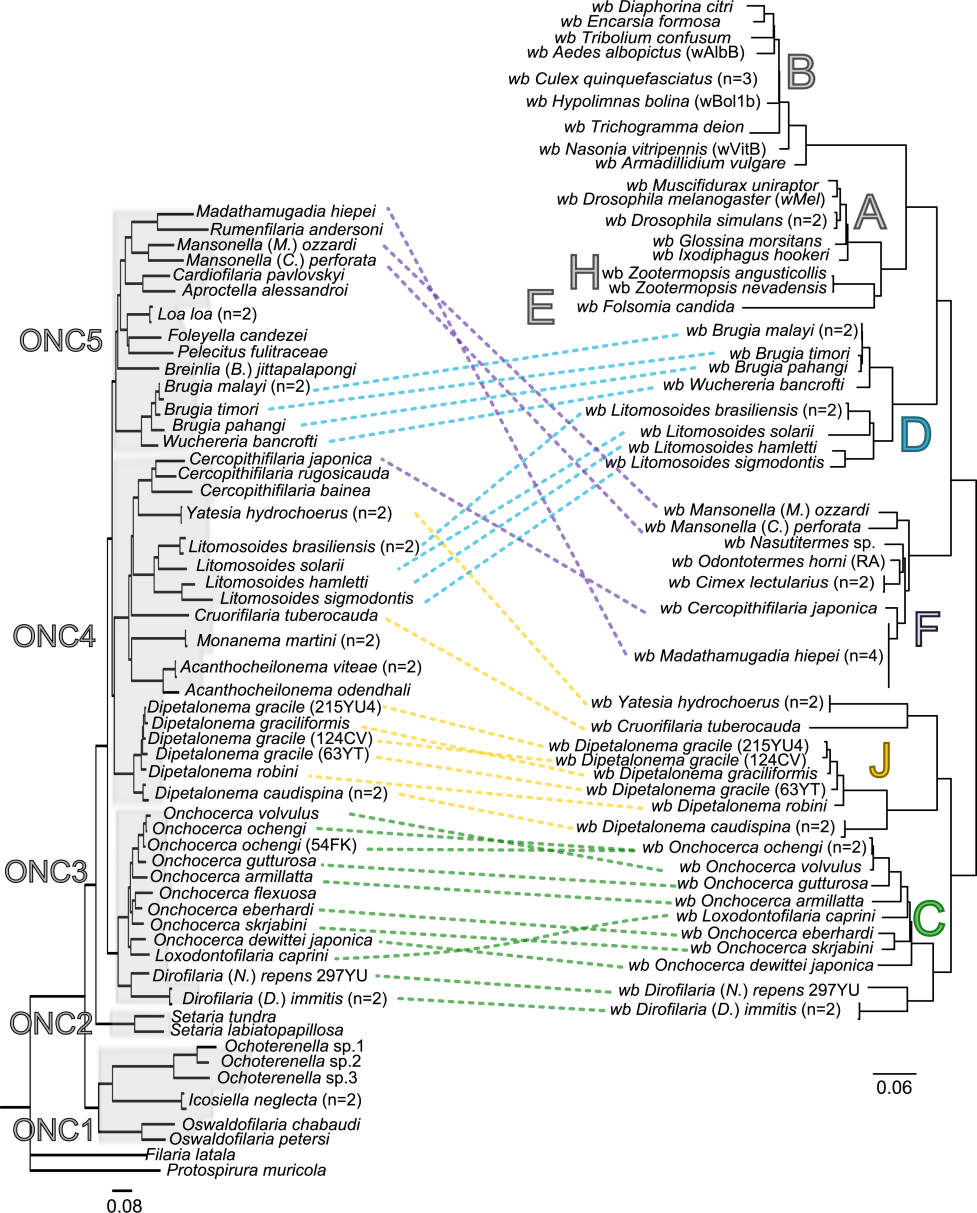
Congruence between filariae and *Wolbachia* phylogenies. The phylogenies of filariae and *Wolbachia* were performed by Maximum Likelihood (ML) inference using RAxML. The scale bar indicates the distance in substitutions per nucleotide. The filariae clades (ONC1-ONC5) were defined according to Lefoulon et al. (2015). The *Wolbachia* supergroups (A–H) were identified according to Werren, Zhang & Guo (1995), Bandi et al. (1998), Lo et al. (2002), and Ros et al. (2009). Abbreviation: *wb*, *Wolbachia*; n, number of studied specimens. Dotted lines show the association between a filarial species and its symbiont. Colors illustrate the bacterial supergroup in filariae.

**Figure 4:**
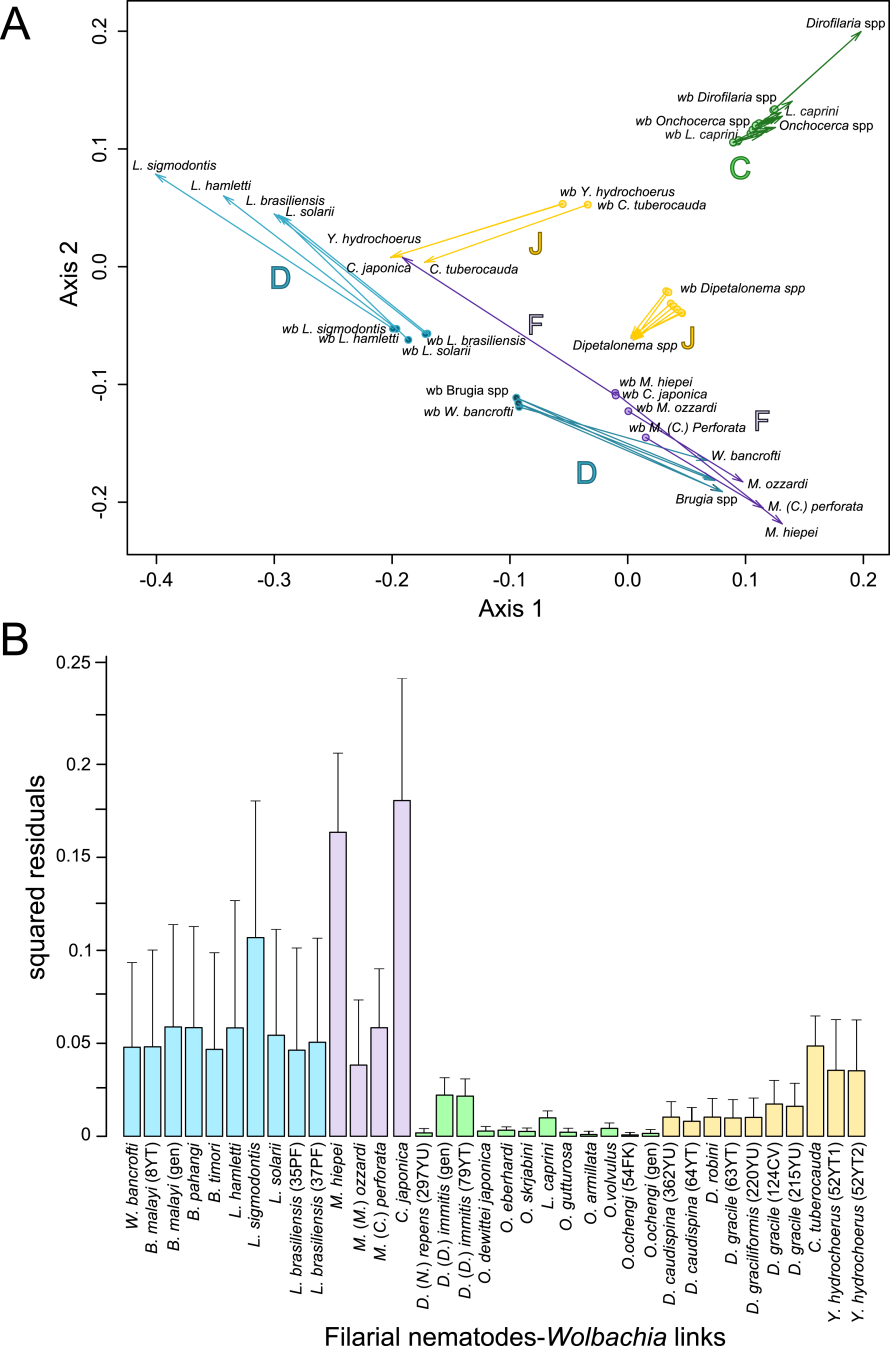
Analysis of coevolution between filariae and *Wolbachia*. A PACo global-fit analysis of *Wolbachia* and their filarial hosts phylogenies was performed. (A) Representative plot of a Procrustes superimposition analysis which minimizes differences between the two partners’ principal correspondence coordinates of patristic distances. For each vector, the start point represents the configuration of *Wolbachia* and the arrowhead the configuration of filarial hosts. The vector length represents the global fit (residual sum of squares) which is inversely proportional to the topological congruence. (B) Contribution of each *Wolbachia*-filariae association to a general coevolution. Each bar represents a Jacknifed squared residual and error bars represent upper 95% confidence intervals. Abbreviation: *wb*, *Wolbachia*.

Since 1998, the hypothesis suggesting that *Wolbachia* is ubiquitous in filariae and co-evolved with them has been commonly accepted (Bandi et al., 1998). However, our recent studies on supergroup F of *Wolbachia* have clearly exposed incongruences with this hypothesis (Ferri et al., 2011; Lefoulon et al., 2012). The key problem in the former studies was that they were based on either a small sampling of *Wolbachia*-infected filarial species and/or a low phylogenetic resolution of the onchocercid evolutionary history. Indeed, the commonly accepted phylogeny of Onchocercidae was erroneous, with distant species considered to be close and vice-versa (Lefoulon et al., 2015). Now, given the 5 strongly supported clades we described within the Onchocercidae (Fig. 1) (Lefoulon et al., 2015), the coevolution of *Wolbachia* and filariae needs to be reassessed. Although a general co-evolutionary pattern was observed (Fig. 3), and the PACo global-fit analysis also indicated a global coevolution between filariae and their *Wolbachia* symbionts (m${}_{XY}^{2}=1.097837$, *p*-value <0,001), distinct local features are apparent. Indeed, the superimposition plot shows 7 groups of filariae-*Wolbachia* associations (Fig. 4A), and the relative contribution of each association to the global fit appears strongly unequal (Fig. 4B). The comparison of both filariae and *Wolbachia* phylogenies revealed strong disparities of the co-phylogenies among the different symbiont supergroups (Fig. 3).

Thus, supergroup C *Wolbachia* and their filarial hosts form one cluster as a result of a strong congruence between their phylogenies (Fig. 4A). Each association in this group presents low squared residual values, which are representative of strong co-evolutionary associations (Fig. 4B). Interestingly, in general, supergroup C *Wolbachia* follows the same evolutionary pattern as that of the ONC3 filarial clade (Fig. 3). Two exceptions can be observed: the position of *Wolbachia* from *Loxodontofilaria caprini* appears not to be congruent, although statistically weakly supported (bootstrap: 42); and *Onchocerca flexuosa* is not infected by *Wolbachia*, which is known to be a secondary loss due to the large number of nuwts in the genome of this species (McNulty et al., 2010).

Supergroup J *Wolbachia* and their filarial hosts are divided in two clusters, one including *Dipetalonema spp.* and their endosymbionts, and the second one including *C. tuberocauda* and *Y. hydrochoerus* and their endosymbionts (Fig. 4A). Within the type J, evolution of *Wolbachia* from *Dipetalonema* species displays a strong congruence with the evolution of their host, unlike *Wolbachia* from *Cruorifilaria* and *Yatesia* (Fig. 4B). Indeed, these two filariae species are not closely related, although they both belong to the same ONC4 clade (Fig. 3). Nonetheless, *C. tuberocauda* and *Y. hydrochoerus* share the same host (*Hydrochoerus hydrochaeris*) and the same neotropical localization as the *Dipetalonema* species; therefore, horizontal transmission is conceivable. Supergroup J *Wolbachia* infect exclusively filariae from ONC4, but this clade also contains five *Wolbachia*-free species: *Acanthocheilonema viteae, A. odendhali, Monanema martini, Cercopithifilaria bainae* and *C. rugosicauda* (Fig. 3). Hence, our results suggest events of horizontal transmission of supergroup J *Wolbachia* in the ONC4 clade, as well as secondary losses.

The supergroups D and F *Wolbachia* and their filarial hosts are each divided into two clusters, supporting an absence of global coevolution between the bacteria and the filariae (Fig. 4). Indeed, the evolution of supergroups D and F are not perfectly congruent with the evolution of their filarial hosts (Fig. 3). Some associations are clearly incongruent with a co-evolutionary hypothesis, such as the relationship between *Wolbachia* and *C. japonica* (supergroup F), *M. hiepei* (supergroup F), *M*. (*C*.) *perforata* (supergroup F), and *L. sigmodontis* (supergroup D) (Fig. 4B). Regarding the *Wolbachia* from supergroup D, one subgroup is distributed in the *Litomosoides* group belonging to the ONC4 clade, and a second group is distributed in the *Brugia*/*Wuchereria* group belonging to the ONC5 clade. The filarial host discontinuity observed in the supergroup D *Wolbachia* suggests that horizontal transfer events occurred between the two filarial host groups. Regarding the supergroup F *Wolbachia*, the bacteria were identified in distant filarial host species (Fig. 3): *Madathamugadia hiepei* and *Mansonella* spp. belonging to the ONC5 clade, and *Cercopithifilaria japonica* belonging to the ONC4 clade. The ONC5 clade also contains 7 *Wolbachia*-free species: *Pelecitus fulitraceae*, *Foleyella candezei, L. loa*, *Breinlia (B.) jittapalapongi*, *Aproctella alessandroi, Cardiofilaria pavlovskyi* and *Rumenfilaria andersoni*. Supergroup F also has the peculiarity of including some bacteria infecting arthropods species (e.g,. *Cimex lectularius* and termite species), supporting one or several horizontal transfers from filariae to arthropods during evolution.

Our current results indicate local patterns of co-evolution and multiple horizontal transmission events of *Wolbachia* within the Onchocercidae family, which are not limited to supergroup F. In addition, supergroup C is the only one to exhibit strong co-speciation with their hosts and not to show any evidence of horizontal transmission events. Recent identification of higher genome plasticity and a lack of synteny within supergroup D compared to supergroup C (Comandatore et al., 2015) supports the uniqueness of supergroup C.

### Origin of *Wolbachia* infection

To date, 26 scientific articles have presented a rooted phylogenetic tree of *Wolbachia* and discuss the evolutionary origin of *Wolbachia* using different methods and a variety of datasets (genes, multi-genes or genomes). The majority of these phylogenetic trees were rooted with *Ehrlichia*, *Anaplasma* or *Rickettsia* as outgroups. Among them, 10 have established the origin of *Wolbachia* between symbionts of arthropods (supergroups A and B) and symbionts of filariae (supergroups C, D, and J) (Baldini et al., 2014; Bandi et al., 1998; Comandatore et al., 2013; Dumler et al., 2001; Fenn & Blaxter, 2006; Ferri et al., 2011; Nikoh et al., 2014; Sironi et al., 1995; Taylor & Hoerauf, 1999; Unver et al., 2003); 4 have indicated a rooting in the symbionts of arthropods, and more specifically between supergroups A and B (Anderson & Karr, 2001; Bordenstein & Rosengaus, 2005; Bordenstein et al., 2009; Giordano, O’Neill & Robertson, 1995; Koutsovoulos et al., 2014); 6 have found a rooting in the symbionts of filariae (Casiraghi et al., 2001a; Casiraghi et al., 2004; Casiraghi et al., 2001b; Czarnetzki & Tebbe, 2004; Dittmar & Whiting, 2004; Rowley, Raven & McGraw, 2004); 2 studies have proposed the symbiont of *Radopholus similis* as a sister group of all other *Wolbachia* (Glowska et al., 2015; Haegeman et al., 2009); and finally 3 studies, including a recent phylogenomic analysis (Casiraghi et al., 2003; Gerth et al., 2014; Ramirez-Puebla et al., 2015), have presented the symbiont of *Folsomia candida* (supergroup E) as a sister group of all other *Wolbachia*.

**Figure 5:**
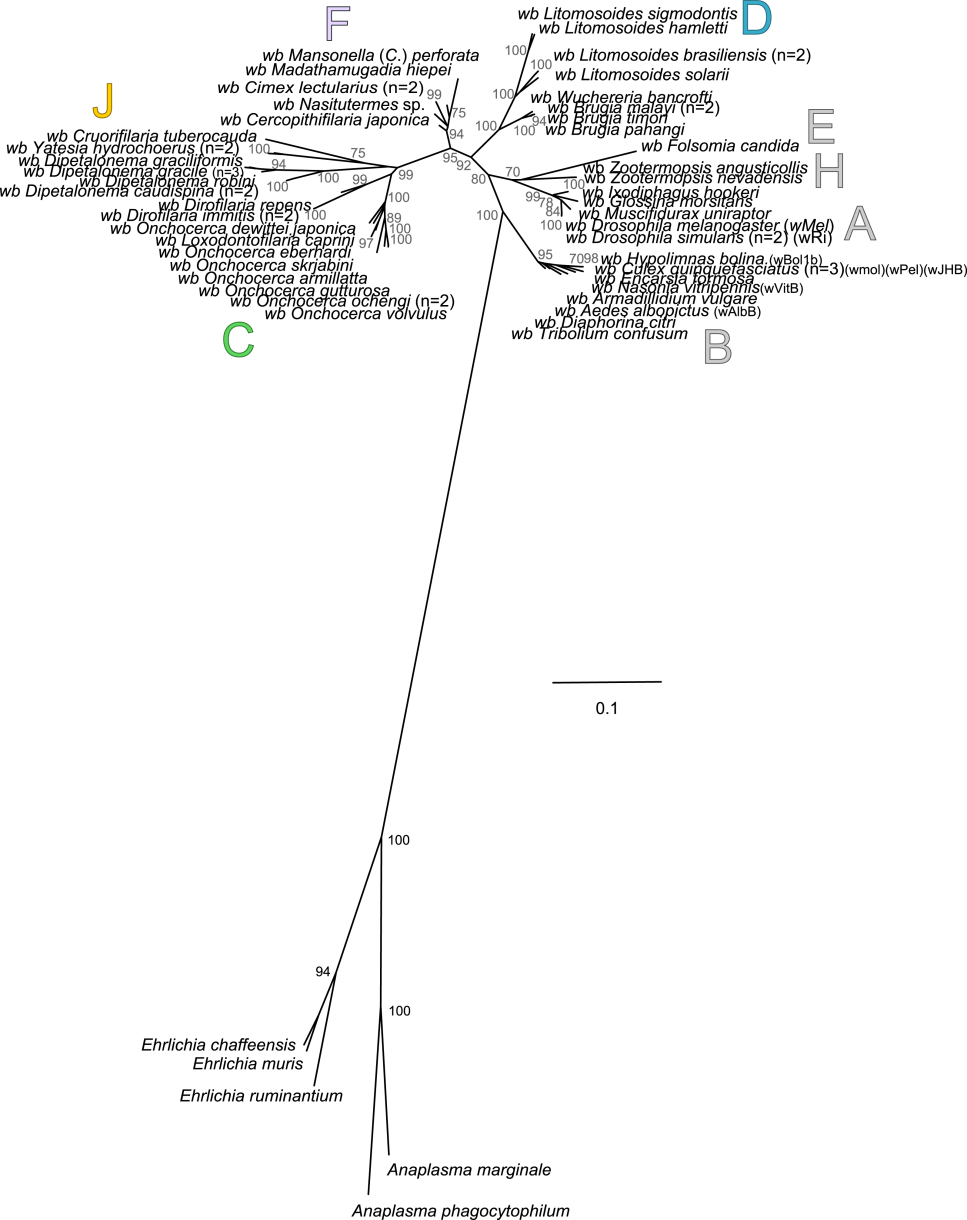
Rooted phylogenetic tree of *Wolbachia* based on 7 markers by Maximum Likelihood. Analysis based on partitioned concatenation of *dnaA*, *groEL*, *ftsZ*, *coxA*, *fbpA* and *gatB* amino acid sequences. The total length of datasets is approximately 1,100 aa. 54 *Wolbachia* strains were analysed. The species *Anaplasma marginale*, *A. phagocytophilum*, *Ehrlichia chaffeensis*, *E. muris* and *E. ruminantium* species were used as outgroups. The topology was inferred using Maximum Likelihood (ML) inference using phyML. Nodes are associated with Bootstrap values based on 500 replicates. The *Wolbachia* supergroups (A–H) were identified according to Werren, Zhang & Guo (1995), Bandi et al. (1998), Lo et al. (2002) and Ros et al. (2009). The scale bar indicates the distance in substitutions per nucleotide. Abbreviation: *wb*, *Wolbachia*; n, number of studied specimens.

Our rooted phylogenetic tree of *Wolbachia* is based on the concatenation of six genes (*groEL*, *ftsZ*, *dnaA*, *gatB*, *fbpA* and *coxA*) and presents the supergroup B of *Wolbachia* as a sister group of all other *Wolbachia* (Fig. 5). However, our analysis also reveals high branch distance between outgroups and ingroups suggesting that the choice of *Ehrlichia* and *Anaplasma* as outgroups induces a long-branch attraction (LBA) artefact due to their high genetic divergence compared to ingroups, as previously suggested (Bordenstein et al., 2009). Moreover, when their outgroups are included in the alignments, substitution saturation was also detected by the Xia method (Xia et al., 2003) for three molecular markers (*coxA*, *p*-value = 0.92; *gatB, p*-value = 0.15; and *dnaA*, *p*-value = 0.83) out of 7 analysed (Table 5). Substitution saturation decreases phylogenetic information contained in the sequences and generates phylogenetic reconstruction which does not reveal correct phylogenetic relationships (Xia & Xie, 2001; Xia et al., 2003). Thus, without identification of accurate outgroups, rooting of *Wolbachia* phylogeny and subsequent analyses are misleading. Consequently, alternative methods need to be investigated.

**Table 5:** Detection of substitution saturation in dataset. Detection of substitution saturation in alignments of *fbpA*, *ftsZ*, *coxA*, 16S rDNA, *gatB*, *groEL*, and *dnaA* markers with and without inclusion of outgroups by the Xia method (2003). “*N*” indicates the number of operational taxonomic units (OTU) simultaneously analysed. “DF” indicates the number of nucleotides considered by the analysis. “ISS” is the index of substitution saturation computed by the Xia method (2003). “ISS.cSym” and “ISS.cAsym” are the theoretical critical substitution saturation index for extreme symmetric or asymmetric topologies, respectively. Saturation is detected if ISS is superior to ISS critical. The difference between indices is compared by a two-tailed *t* test, and the *p*-values are indicated for each alignment.

Markers	*N*	DF	ISS	ISS.cSym	*p*-value	ISS.cAsym	*p*-value
*fbpA*	32	462	0.244	0.698	0	0.372	0
*fbpA*+ outgp	32	461	0.281	0.698	0	0.371	0.0002
*ftsZ*	32	515	0.177	0.704	0	0.378	0
*ftsZ*+ outgp	32	518	0.246	0.704	0	0.378	0
*coxA*	32	488	0.274	0.701	0	0.376	0.0001
*coxA*+ outgp	32	489	0.378	0.701	0	0.376	0.9229
16S rDNA	32	1,255	0.339	0.763	0	0.471	0
16S rDNA + outgp	32	1,251	0.327	0.763	0	0.471	0
*gatB*	32	564	0.251	0.709	0	0.381	0
*gatB*+ outgp	32	558	0.338	0.708	0	0.38	0.155
*groEL*	32	745	0.13	0.727	0	0.405	0
*groEL*+ outgp	32	836	0.267	0.735	0	0.419	0
*dnaA*	32	407	0.259	0.692	0	0.363	0
*dnaA*+ outgp	32	410	0.357	0.692	0	0.364	0.8301

**Notes.**

Abbreviation Outgp outgroups

The current phylogenies of both *Wolbachia* and their filarial hosts allow us to attempt an explanation of the symbiotic history on the basis of the most parsimonious co-phylogeny scenario. Our data raise the hypothesis that *Wolbachia* infection in the onchocercid family was acquired between the diversification of ONC2 (Setarinae) and the later derived clades (Fig. 3). Phylogenetic and co-evolutionary data suggest that supergroup C has diverged in the ONC3 filarial clade, which is the first diverged clade from ONC2 (Fig. 3). These data imply that supergroup C of *Wolbachia* would be the ancestral symbionts. All other alternatives involve numerous horizontal transfers of *Wolbachia* within the Onchocercidae. Upon agreement on the basal position of supergroup C in the co-phylogeny of *Wolbachia* and filariae, a “walking” method can be used to analyse the other bacteria supergroups (Fig. 3): supergroup J is thus the closest to supergroup C, infecting filarial species in ONC4 clade; subsequently, supergroups F and D appear, infecting respectively both ONC4 and ONC5 and showing horizontal transfers and losses, but also local coevolution.

**Figure 6:**
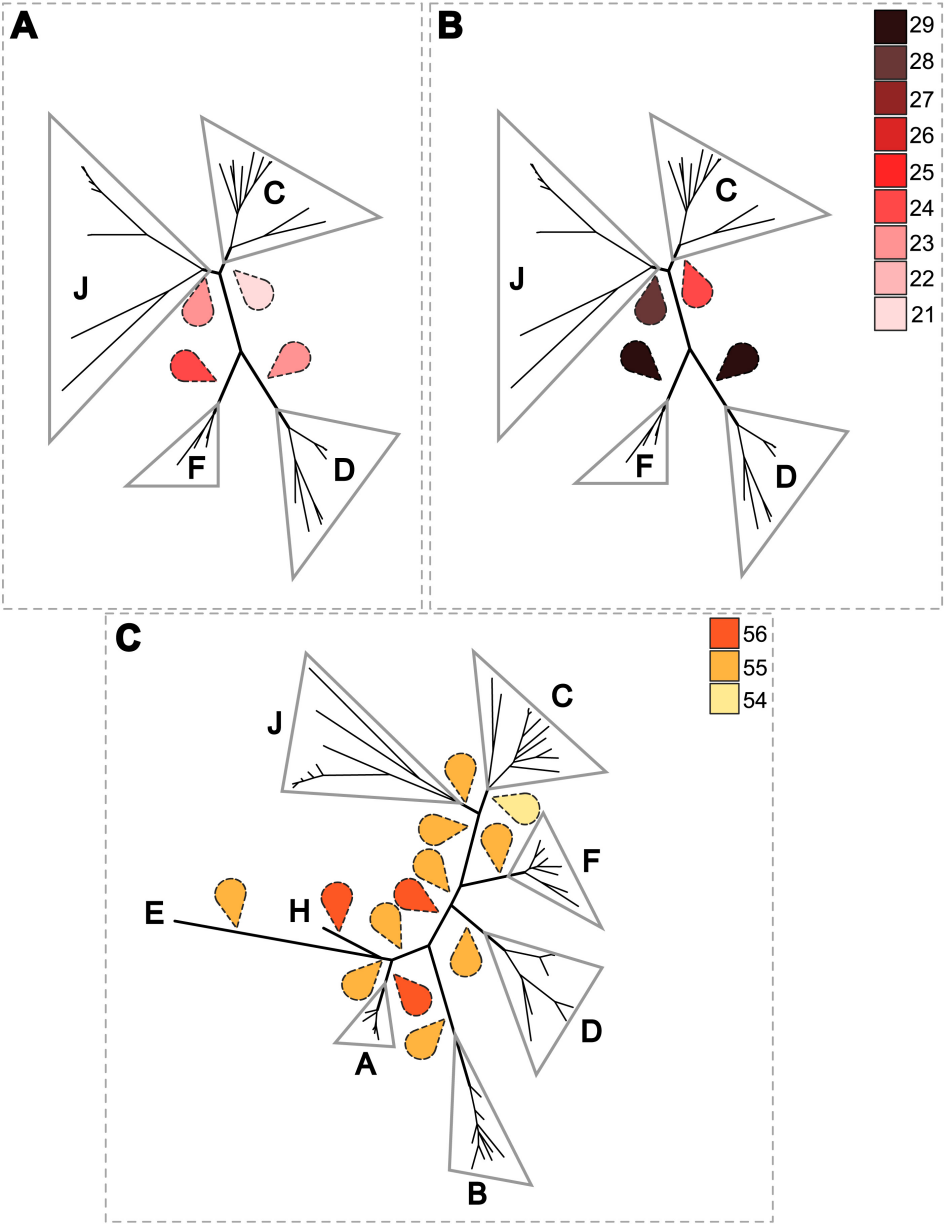
Estimation of the cost of co-evolutionary scenarios based on different rooted *Wolbachia* phylogenies. An event-cost method with Jane software was performed. Representations of the costless co-evolutionary reconstructions between *Wolbachia* and their hosts are shown using the following regime cost criteria: co-speciation = 0; duplication = 1; loss = 1; and duplication and host switch = 2. Different roots for the phylogeny of *Wolbachia* were tested. The colored drop tips indicate the examined rooting and the color represents the lowest associated cost. (A) co-evolutionary reconstruction between 36 *Wolbachia* and their filarial hosts without time constraint. (B) co-evolutionary reconstruction between 36 *Wolbachia* and their filarial hosts with time constraint. (C) co-evolutionary reconstructions between 60 *Wolbachia* and their filarial and arthropod hosts.

To further analyse the coevolution scenarios, an event-based method using Jane v4 (Conow et al., 2010) was applied to our data, computing each scenario while minimizing the cost of evolutionary events. Focusing on *Wolbachia* from filariae reveals that a rooting of the phylogeny of *Wolbachia* within supergroup C represents the co-evolutionary scenario associated with the lowest cost (Figs. 6A and 6B). Cost differences between different *Wolbachia*-rooted tree analyses are increased if a time constraint is applied to limit host switch events which could be inconsistent with filarial phylogeny (see Material and Method) (Figs. 6A and 6B). Similarly, the analysis including *Wolbachia* from arthropods also suggests a rooting of the phylogenetic tree of *Wolbachia* within supergroup C because of its lowest co-evolutionary scenario cost, even if cost differences are weak (Fig. 6C). Unfortunately, it would be difficult to increase these cost differences by defining time zones, as for analysis performed with the *Wolbachia* from filariae (see Material and Method). Indeed, timescale evolution of filariae is unknown due to absence of paleontological data, so it is not possible to position their speciation *versus* the evolutionary history of arthropods. The two proposed analyses using Jane have limitations (such as time zone definition), but all results suggest that supergroup C is the ancestrally derived group of nematode *Wolbachia* symbionts. This scenario may lead to the hypothesis that *Wolbachia* have primarily infected and diverged within the filarial species and then were transferred from the supergroup D *Wolbachia* to arthropod species.

Such a hypothesis on the origin of *Wolbachia* does not simply change the reading of the phylogenetic tree, but may also improve our understanding of *Wolbachia* adaptation to their hosts at the evolutionary scale. Indeed, the genome structures of *Wolbachia* infecting arthropods are relatively large and plastic, including numerous mobile elements (repeated elements, transposons, and phages); whereas symbionts of filariae present more compact genomes without actively mobile DNA. Thus, the genomic structure could be regarded differently, as the acquisition of plasticity and increased insertions of mobile DNA may be patterns linked to a change of host range. Recently, analyses of complete genomes of *Wolbachia* indicated that symbionts belonging to supergroup C from *Onchocerca ochengi* (*wOo*) and *Dirofilaria immitis* (*wDi*), present specific genomic features including reduced genome size, a low number of genomic rearrangements, fewer insertion sequences, and losses or pseudogenization of numerous genes (e.g., 88 unique losses for *wOo*) (Comandatore et al., 2015; Darby et al., 2012). These data would support a reductive evolution of supergroup C (Darby et al., 2012). However, it was also suggested that such features are characteristic of endosymbiotic bacteria with long-lasting relationships with their hosts (Comandatore et al., 2015), which is consistent with our results showing that supergroup C is strongly co-evolved with its hosts. Nevertheless, we cannot rule out that *Wolbachia* could have originally had a very plastic genome structure and reductive evolution may have been promoted by strong coevolution with their hosts.

In conclusion, we show that patterns of coevolution between *Wolbachia* and their filarial hosts are much more complex than previously assumed, with numerous examples of secondary losses of symbionts and horizontal transfers between clades. Strict coevolution is restricted to filarial clade ONC 3 with *Wolbachia* in supergroup C. Moreover, since tree-rooting options are contentious for *Wolbachia*, we have applied a series of novel analyses to address the question of the origin of the *Wolbachia* symbiosis. These methods, although not without their own limitations, place the origin within supergroup C rather than an arthropod-specific supergroup.

## Supplemental Information

10.7717/peerj.1840/supp-1 File S1 List of author(s) and dates of studied species List of author(s) and dates associated with name of filariae, their vertebrate hosts or arthropods.

10.7717/peerj.1840/supp-2 Figure S1 *Wolbachia* immunostaining in four female specimens belonging to the *Dipetalonema* genus Sections stained with a rabbit polyclonal antiserum against *Wolbachia* surface protein (WSP) of *Brugia pahangi Wolbachia* (Wol-Bp-WSP, dilution 1:2,000). Presence of *Wolbachia* (small red dots) is indicated by an arrow. (A–B) a *Dipetalonema caudispina* specimen (64YT) presents staining in the hypodermal lateral chord. (C–D) a *Dipetalonema robini* specimen (217YU) presents staining in the hypodermal lateral chord and germline (not shown). (E–F) a *Dipetalonema gracile* specimen (215YU) presents staining in the hypodermal lateral chord and reproductive tract. (G–H) a *Dipetalonema graciliformis* specimen (220YU) presents staining in the hypodermal lateral chord and reproductive tract. Legend: I, intestine; U, uterus; c, cuticle; h, hypodermal lateral chords; m, muscles; hypodermal lateral chord delimited by stars; * indicated lateral plan.

10.7717/peerj.1840/supp-3 Figure S2 *Wolbachia* immunostaining in female specimens belonging to *Cruorifilaria*, *Litomosoides* and *Cardiofilaria* genera Sections stained with a rabbit polyclonal antiserum against *Wolbachia* Surface Protein (WSP) of *Brugia pahangi Wolbachia* (Wol-Bp-WSP, dilution 1:2,000). Presence of *Wolbachia* (small red dots) is indicated by an arrow. (A–B) a *Cruorifilaria tuberocauda* specimen (57YT) presents staining in the hypodermal lateral chord and reproductive trac. (C–D) a *Litomosoides brasiliensis* specimen (35PF) presents staining in the hypodermal lateral chord and reproductive tract (not shown). (E) a *Litomosoides solarii* specimen (213YU) presents staining in the hypodermal lateral chord and reproductive tract. (F) a *Cardiofilaria pavlovkyi* specimen (180YU) presents staining in the hypodermal lateral chord, reproductive tract and intestinal wall cells. Legend: I, intestine; U, uterus; c, cuticle; h, hypodermal lateral chords; m, muscles; Hypodermal lateral chord delimited by stars; ∗ indicated lateral plan.

10.7717/peerj.1840/supp-4 Table S1 Ethical statement on vertebrate hosts Vertebrate host are indicated in the first column and their collection place is given in the second column. The name of the collected filarial species is given in the third column. Columns 4 and 5 present ethical information on the host collection, i.e., the capture permit number and the ethical committee or legal entity for scientific procedures; columns 6 to 8 describe non-scientific procedures in which filariae were recovered from vertebrates after hunting, necropsies in slaughter houses, or post-veterinary or post-medical procedures. The number of MNHN collection registration is listed in the last column.

10.7717/peerj.1840/supp-5 Table S2 Primers and PCR programs used in this study Abbreviations: size, amplicon size; step 1, denaturation; step 2, annealing; step 3, elongation; T, temperature (°C); D, duration (sec); N, number of cycles; pr., primers; BSA, bovine serum albumin; ref, reference. ∗ Indicates that the primers were designed for nested PCR. References: PS means newly designed for the present study.

10.7717/peerj.1840/supp-6 Table S3 Accession number list of *Wolbachia* from filarial species or from arthropods, and outgroupsused for phylogenetic analyses The name of the species are given in the first column. The identification number of samples are given in the second column. Columns 3 to 9 present accession numbers for each analysed gene: 16S rDNA; 60 kDa chaperonin *groEL* gene; *ftsZ* gene for cell division protein; *dnaA* gene for chromosomal replication initiator protein; cytochrome c oxidase subunit I (*coxA*) gene; putative fructose-bisphosphate aldolase (*fbpA*) gene; and glutamyl-tRNA(Gln) amidotransferase subunit B (*gatB*) gene. Abbreviations: ext. indicates external samples.

10.7717/peerj.1840/supp-7 Table S4 Summary of reports of the presence of *Wolbachia* in filariae Filariae examined in the current study are indicated in bold. Abbreviation: EM, transmission electron microscopy; IM, immunostaining; +, detection of *Wolbachia*; ND, not determined.

## Additional Information and Declarations

### Field Study Permissions

The following information was supplied relating to field study approvals (i.e., approving body and any reference numbers): All information is indicated in Table S1. - Marine Mammal Protection Act; 14327 NMML. - Universidad Nacional Agraria La Molina Ethical Committee; WGS84. - Ethical committee of the Museum National d’Histoire Naturelle; EU Directive; Comitee Cuvier Licence: 68-002; DDCSPP: No. C75-05-15. - BESK; 55/17.04.2006. - CNRS; Prob-DOM-06-PCVI-0007. - National Agriculture and Forestry Research Institute; BDIV 012 CERoPath. - Ministerio del Ambiente de la Republica Bolivariana de Venezuela; Nu 33/2007. - Brazilian Institute of the Environment and Renewable Resources; 261/05, NUFAS/MG-IBAMA. - Peru: local hunting (food collection). - Venezuela: national hunting (population regulation + food collection).

### DNA Deposition

The following information was supplied regarding the deposition of DNA sequences: GenBank Data Library: KU255197 to KU255395.

### Data Availability

The following information was supplied regarding data availability: TreeBase: http://purl.org/phylo/treebase/phylows/study/TB2:S19008.
