# Supplementary material for: Breakdown of coevolution between symbiotic bacteria Wolbachia and their filarial hosts
Source: PeerJ. 2016 Mar 28;4:e1840. doi: 10.7717/peerj.1840 (PMC4824920; doi:10.7717/peerj.1840)
Supplement: File S1 — List of author(s) and dates associated with name of filariae, their vertebrate hosts or arthropods. [file peerj-04-1840-s001.pdf]

Supplementary files : Species and Authorities

Nematodes:

Oswaldofilaria chabaudi Pereira, Souza and Bain, 2010

Oswaldofilaria petersi Bain and Sulahian 1974

Icosiella neglecta (Diesing, 1851)

Setaria labiatopapillosa (Alessandrini, 1848)

Setaria tundra Bain, 1974

Dirofilaria (*Dirofilaria*) *immitis* (Leidy, 1856)

Dirofilaria (*Nochtiella*) *repens* Railliet and Henry, 1911

Foleyella candezei (Fraipont, 1882)

Loa loa (Cobbold, 1864)

Pelecitus fulicaeatrae (Diesing, 1861)

Aproctella alessandroi Bain, Petit, Kosek and Chabaud, 1981

Cardiofilaria pavlovskyi Storm, 1937

Madathamugadia hiepei Hering-Hagenbeck, Boomker, Petit, Killick-Kendrick and Bain, 2000

Rumenfilaria andersoni Lankester and Snider, 1982

Acanthocheilonema odendhali (Perry, 1967)

Acanthocheilonema viteae (Krepkogorskaya, 1933)

Breinlia (*Breinlia*) *jittapalapongi* Veciana, Bain, Morand, Chaisiri, Douanghoupha, Miquel and Ribas, 2015

Brugia malayi (Brug, 1927)

Brugia pahangi (Buckley and Edeson, 1956)

Brugia timori Partono, 1977

Cercopithifilaria bainae Almeida and Vicente, 1984

Cercopithifilaria rugosicauda (Böhm and Supperer, 1953)

Cruorifilaria tuberocauda Eberhard, Morales and Orihel , 1976

Dipetalonema caudispina (Molin, 1858)

Dipetalonema gracile (Rudolphi, 1809)

Dipetalonema graciliformis (Freitas, 1964)

Dipetalonema robini Petit, Bain and Roussilhon, 1985

Litomosoides brasiliensis Lins de Almeida, 1936

Litomosoides hamletti Sandground, 1934

Litomosoides sigmodontis Chandler, 1931

Litomosoides solarii Guerrero, Martin, Gardner and Bain, 2002

Loxodontofilaria caprini Uni and Bain, 2006

Mansonella (*Cutifilaria*) *perforata* Uni, Bain and Takaoka, 2004

Mansonella (*Mansonella*) *ozzardi* (Manson, 1897)

Monanema martini Bain, Bartlett and Petit, 1986

Onchocerca armillata Railliet and Henry, 1909

Onchocerca dewittei japonica Uni, Bain and Takaoka, 2001

Onchocerca eberhardi Uni and Bain, 2007

Onchocerca gutturosa Neumann, 1910

Onchocerca ochengi Bwangamoi, 1969

Onchocerca skrjabini Ruklyadev, 1964

Yatesia hydrochoerus (Yates, 1980)

Filaria latala Chabaud and Mohammad, 1989

Protospirura muricola Gedoelst, 1916

Vertebrate hosts:

Agama agama (Linnaeus, 1758)

Arvicanthis niloticus (Geoffroy,1803)

Ateles E. Geoffroy, 1806

Ateles paniscus (Linnaeus, 1758)

Bos taurus Linnaeus, 1758

Callorhinus ursinus (Linnaeus, 1758)

Canis familiaris Linnaeus, 1758

Capreolus capreolus (Linnaeus, 1758)

Carollia perspicillata (Linnaeus, 1758)

Cebus apella (Linnaeus, 1758)

Cebus olivaceus Schomburgk, 1848

Cervus nippon Temminck, 1838

Chondrodactylus turneri (Gray, 1864)

Crocodilurus amazonicus (spix, 1825)

Glossophaga soricina Pallas, 1766

Gorilla gorilla Savage, 1847

Homo sapiens Linnaeus, 1758

Hydrochoerus hydrochaeris (Linnaeus, 1766)

Ixodes ricinus (Linnaeus, 1758)

Lagothrix poeppigii Schinz, 1844

Meriones unguiculatus (Milne-Edwards, 1867)

Naemorhedus crispus (Robert Swinhoe, 1870)

Oriolus oriolus (Linnaeus, 1758)

Panthera leo (Linnaeus, 1758)

Pelophylax kl. esculentus (Linnaeus, 1758) (syn. *Rana esculenta*)

Pelophylax ridibundu (Pallas ,1771) (syn. *Rana ridibunda*)

Phyllomedusa bicolor (Boddaert, 1772)

Podiceps nigricollis, Brehm, 1831

Rangifer tarandus (Linnaeus, 1758)

Rattus tanezumi Temminck, 1844

Rhinella granulosa (Spix, 1824)

Rhinella marina (Linnaeus, 1758)

Saimiri sciureus (Linnaeus, 1758)

Saltator similis D'Orbigny & Lafresnaye, 1837

Sus scrofa leucomystax Temminck, 1842

Trachops cirrhosis (Spix, 1823)

Tropidurus torquatus (Wied-Neuwied, 1820)

Arthropods :

Cimex lectularius Linnaeus, 1758

Drosophila simulans Alfred Henry Sturtevant, 1919

Ixodiphagus hookeri (Howard, 1908)

Folsomia candida Willem, 1902
