# Supplementary figures and images for: Breakdown of coevolution between symbiotic bacteria Wolbachia and their filarial hosts
Source: PeerJ. 2016 Mar 28;4:e1840. doi: 10.7717/peerj.1840 (PMC4824920; doi:10.7717/peerj.1840)

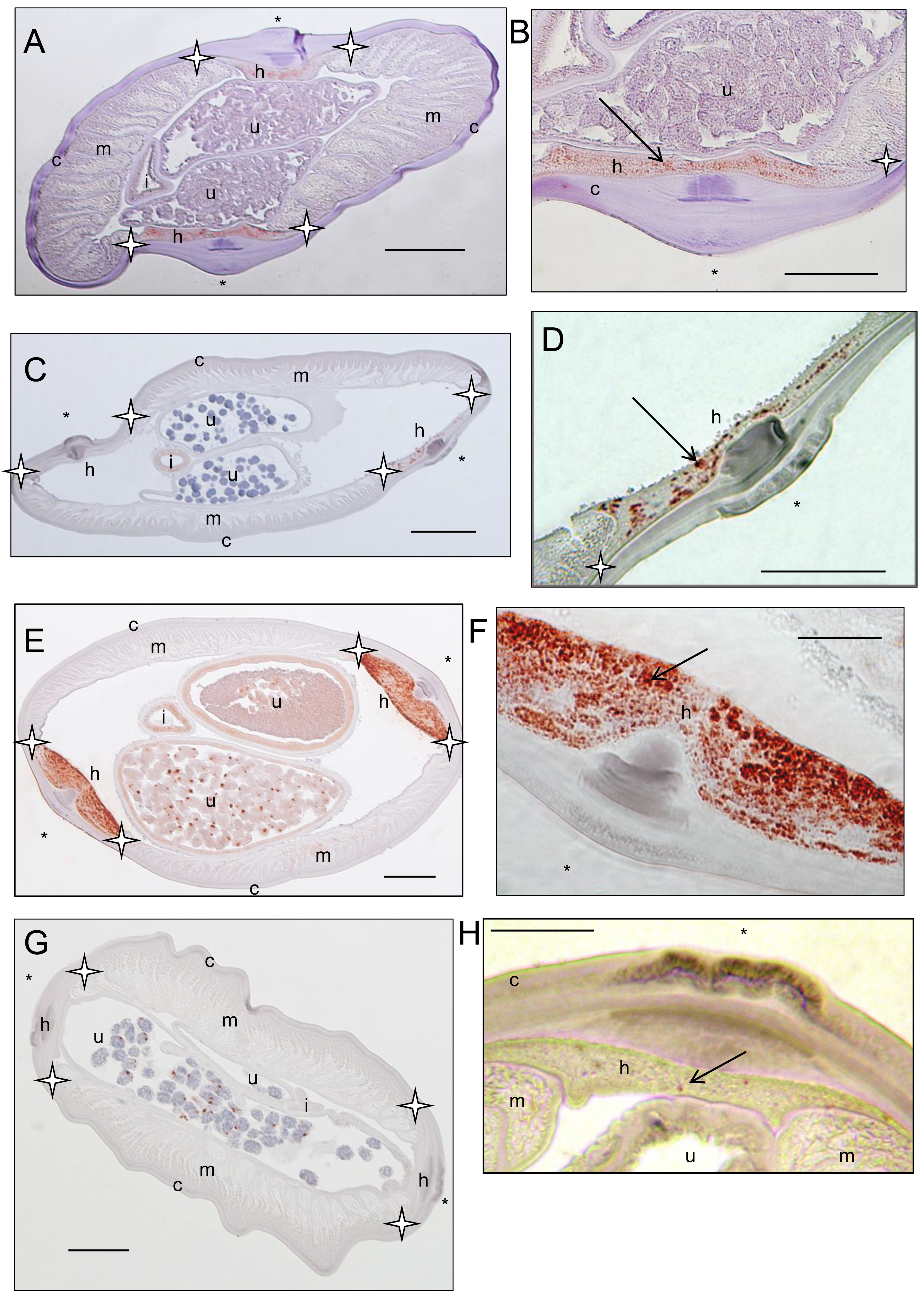

Supplement: Figure S1 — Sections stained with a rabbit polyclonal antiserum against Wolbachia surface protein (WSP) of Brugia pahangi Wolbachia (Wol-Bp-WSP, dilution 1:2,000). Presence of Wolbachia (small red dots) is indicated by an arrow. (A–B) a Dipetalonema caudispina specimen (64YT) presents staining in the hypodermal lateral chord. (C–D) a Dipetalonema robini specimen (217YU) presents staining in the hypodermal lateral chord and germline (not shown). (E–F) a Dipetalonema gracile specimen (215YU) presents staining in the hypodermal lateral chord and reproductive tract. (G–H) a Dipetalonema graciliformis specimen (220YU) presents staining in the hypodermal lateral chord and reproductive tract. Legend: I, intestine; U, uterus; c, cuticle; h, hypodermal lateral chords; m, muscles; hypodermal lateral chord delimited by stars; * indicated lateral plan. [file peerj-04-1840-s002.jpg]

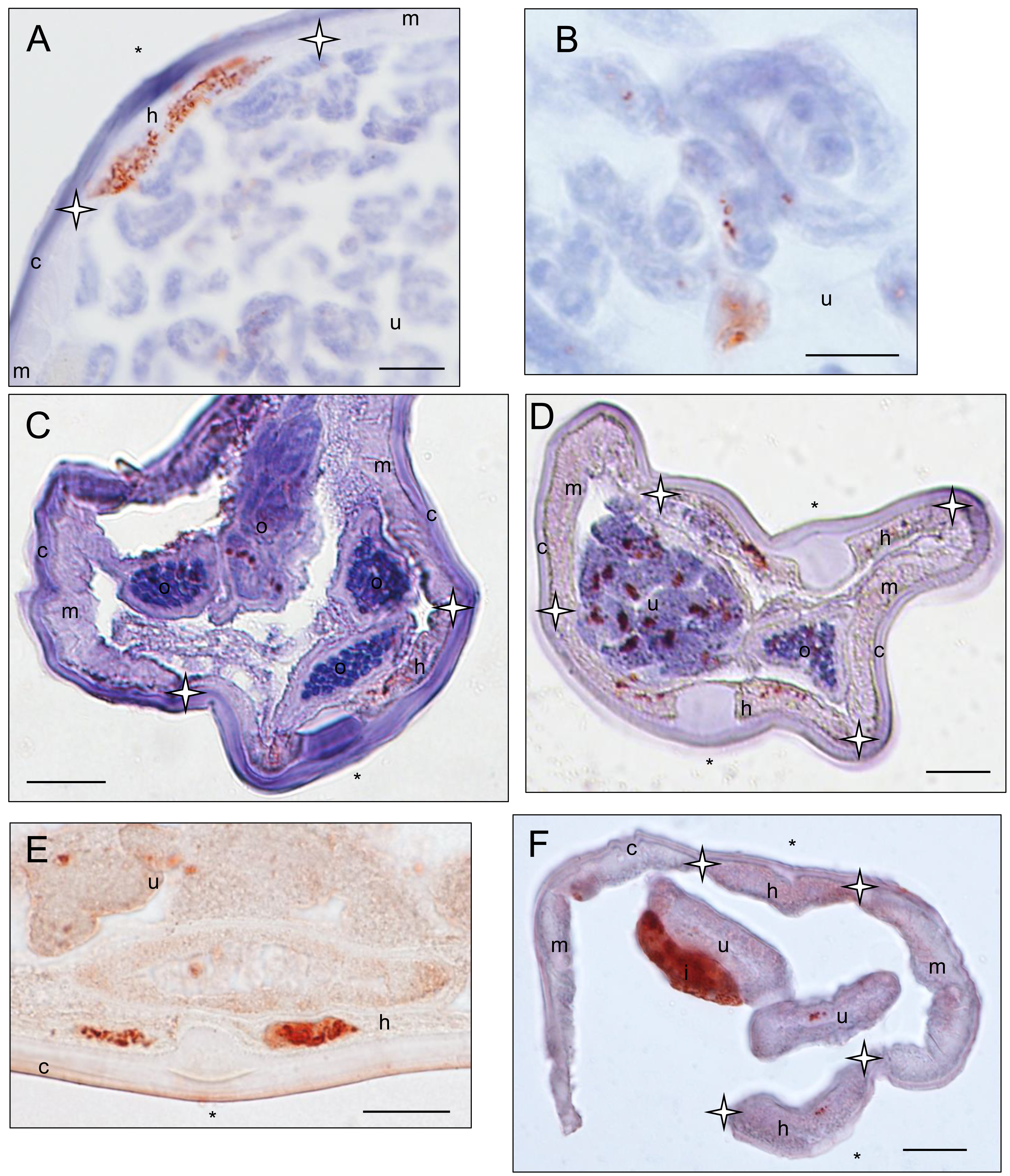

Supplement: Figure S2 — Sections stained with a rabbit polyclonal antiserum against Wolbachia Surface Protein (WSP) of Brugia pahangi Wolbachia (Wol-Bp-WSP, dilution 1:2,000). Presence of Wolbachia (small red dots) is indicated by an arrow. (A–B) a Cruorifilaria tuberocauda specimen (57YT) presents staining in the hypodermal lateral chord and reproductive trac. (C–D) a Litomosoides brasiliensis specimen (35PF) presents staining in the hypodermal lateral chord and reproductive tract (not shown). (E) a Litomosoides solarii specimen (213YU) presents staining in the hypodermal lateral chord and reproductive tract. (F) a Cardiofilaria pavlovkyi specimen (180YU) presents staining in the hypodermal lateral chord, reproductive tract and intestinal wall cells. Legend: I, intestine; U, uterus; c, cuticle; h, hypodermal lateral chords; m, muscles; Hypodermal lateral chord delimited by stars; ∗ indicated lateral plan. [file peerj-04-1840-s003.jpg]
